# The American Journal and Library of Dental Science

**Published:** 1845-09

**Authors:** 


*The American Journal and Library of Dental Science.*—This publication may now be regarded as established upon a firm basis. It has overcome the difficulties with which it has hitherto had to contend, and they have neither been few nor small, and though it may have to encounter others, its prospects for the future are more flattering than they were during the three or four first years of its existence. It has required, all along, the most constant and untiring efforts to sustain it, but it now has a list of subscribers sufficiently large, provided all would pay punctually, to defray the expenses of publication. Most of those who take it, pay their subscriptions promptly and cheerfully, but a few have been exceedingly remiss with regard to this matter, and we do hope they will be less negligent for the future. The importance of complying with the terms of subscription, will be apparent to every one, when we mention the fact, that there is now due the Journal, on back subscriptions, upwards of thirteen hundred dollars. We hope, therefore, all who are in arrears, will, on the receipt of this number, send us at once, by letter, the amount of their indebtedness, and the subscription for the present volume. If all will do this, it will save us a vast amount of trouble and perplexity.

A few have complained that the subscription is too high, and have said we ought not to charge more than three dollars per annum for it. In reply to such, we say we would be as glad to issue it at three dollars, as they would to receive it at that price; but our subscription at present, will not enable us to do it. It costs as much to publish the Journal as is received for it from subscribers. It cannot be expected that a periodical having as limited a circulation as this, should be published at as low a price as a medical journal, which may obtain from one to five thousand subscribers. Although five dollars for a work no larger than this may seem a high price, it should be recollected, that the books which have been re-published in it, to say nothing of the original articles, could not be procured for double the amount. Some of them, in fact, are out of print, and not to be had, separately from the Journal, at any price.

With regard to the benefits that have resulted from this publication, it does not become us to speak; but the advantages of a medium of intercommunication between the members of the profession, through which discoveries and improvements may be made known and available to all, and theories and principles discussed and settled, every one will acknowledge to be great. To establish such a medium of intercommunication was the object of the projectors of the Journal, and to perpetuate it, has been, and is, the object of the association whose organ and property it now is. In the management of it, the editors have endeavored to make it as valuable and useful to the profession as possible. For the approval which their labors have received from so many of their professional brethren, both in this country and Europe, they feel grateful; and hope still to be able to merit their approbation, by redoubling their efforts to make it more worthy of their favorable regard.

